# A Rare Case of Primary Adenocarcinoma of the Eyelid: Case Presentation and Review of Literature

**DOI:** 10.7759/cureus.16580

**Published:** 2021-07-23

**Authors:** Noha N Soror, Innocent Lutaya, Parth Shah, Lori Hemrock, Robert Bennett, Gary Gibson

**Affiliations:** 1 Internal Medicine, Western Reserve Health Education/Northeast Ohio Medical University, Warren, USA; 2 Internal Medicine, American University of Antigua, Warren, USA; 3 Medical Oncology, The Hope Center for Cancer Care, Warren, USA; 4 Pathology, Steward Health Care, Warren, USA; 5 Internal Medicine, Steward Health Care, Warren, USA

**Keywords:** primary cutaneous mucinous carcinoma, primary mucinous carcinoma of skin, nonmelanoma skin cancer, malignant tumors of the eyelid, eyelid mass

## Abstract

Primary mucinous adenocarcinoma (PMA) of the eyelid is a very rare malignancy with an incidence of 0.07 per million. Most cases are elderly males with an indolent course of local growth over months. We report a rare case of a 61-year-old gentleman with an aggressive course of PMA. The patient presented with a painless lower right eyelid swelling that developed over a four-month period. Incisional biopsy of the mass disclosed mucinous adenocarcinoma, positive for cytokeratin (CK)7 but negative for thyroid transcription factor 1, S100, and CK20 expression. Magnetic resonance imaging of the orbits revealed an enhancing infiltrative mass extending from the right lower eyelid to the medial canthus and posteriorly into the orbit, the right ethmoid sinuses, and extraconal fat within the orbit. Workup for metastatic disease including scans of chest, abdomen, and pelvis as well as positron emission tomography/computed tomography were negative for other masses. The patient underwent extensive surgery that included exenteration of the right orbit and cervical lymph node dissection followed by adjuvant radiation therapy and chemotherapy due to the extent of periorbital tumor invasion of contiguous tissues. Diagnosis of PMA is a clinical challenge, and immunohistochemistry is essential for diagnosis. To confirm it as a primary tumor, exclusion of metastasis from elsewhere is appropriate. Reported treatment modalities include Mohs micrographic surgery or excision with frozen section and safety margin. Exenteration of the orbit may be indicated depending on the extent of orbital invasion by the tumor. There is limited evidence to guide treatment and follow-up, with information consisting mostly of published case reports and case series.

## Introduction

Primary mucinous adenocarcinoma (PMA) of the eyelid is a rare adnexal neoplasm that originates from eccrine sweat glands [1]. It tends to have a higher incidence in Caucasian males. The median age at diagnosis is 60 years [2]. The lesions usually present as slowly growing painless nodules [3]. Metastasis is a rare sequel of PMA, even though, the rate of local recurrence after surgical excision is high [4]. We report the case of a 61-year-old male with a PMA of the right lower eyelid who had extensive local invasion and cervical lymph node metastasis that mandated aggressive surgical management followed by adjuvant chemotherapy and radiation therapy.

## Case presentation

**Figure 1:**
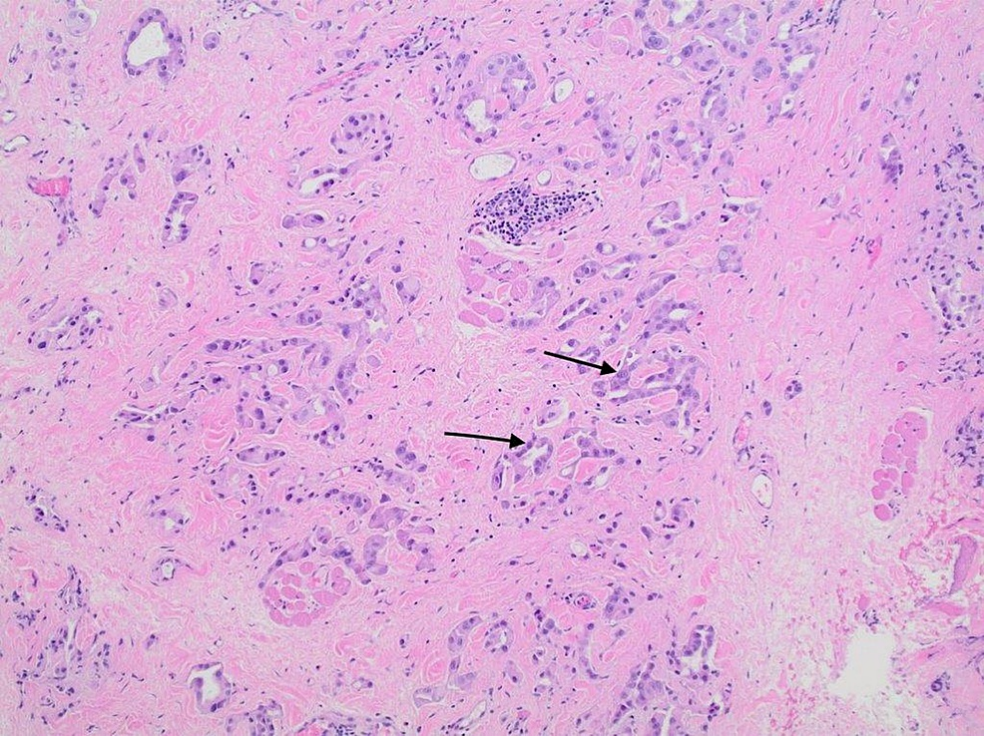
Infiltrating glands and cords of malignant cells in a background of benign fibrovascular tissue.

**Figure 2:**
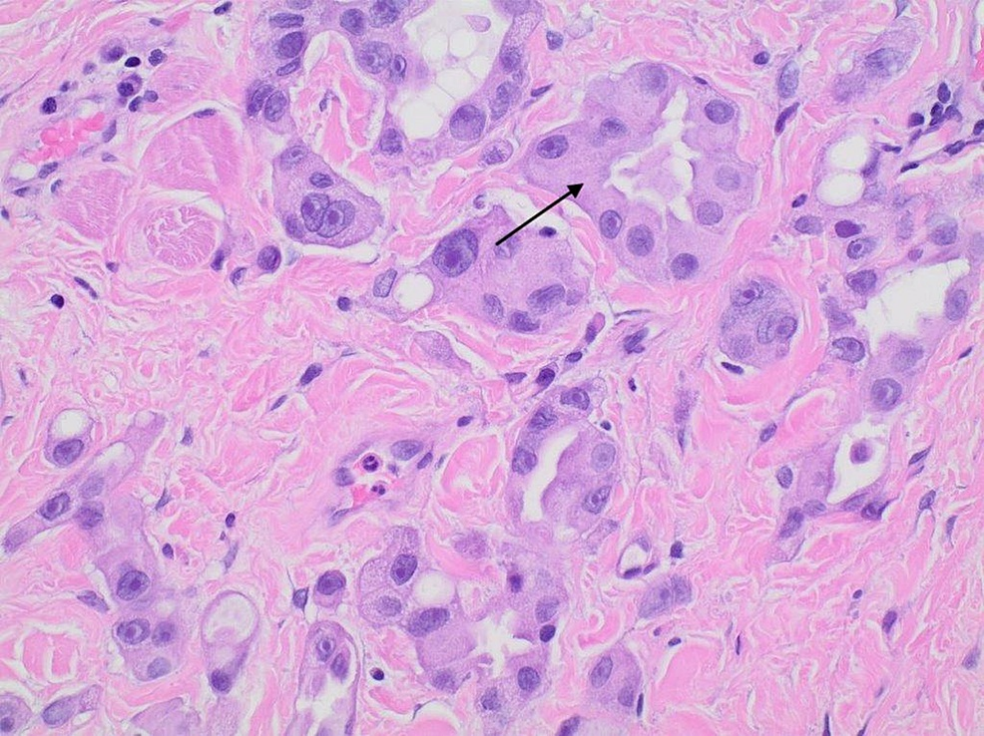
Higher magnification showing small nests and glandular structures.

**Figure 3:**
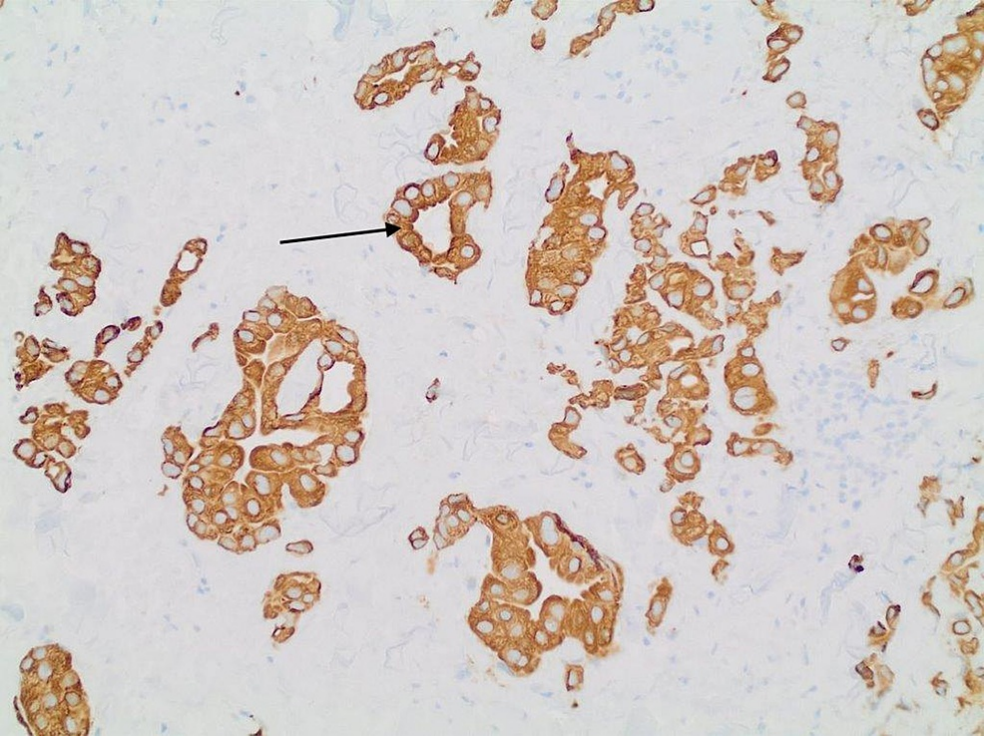
CK7 positive staining of the right lower eyelid biopsy. CK7: cytokeratin 7

**Figure 4:**
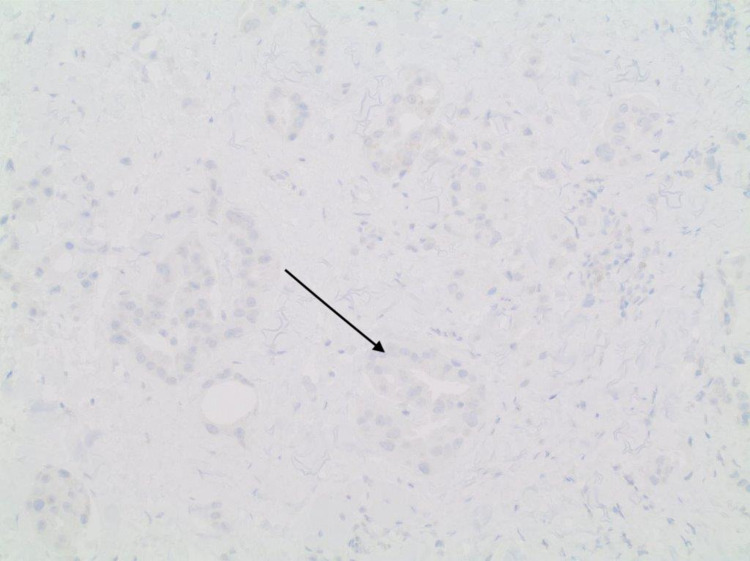
Negative immunostaining for TTF-1. TTF-1: thyroid transcription factor 1

**Figure 5:**
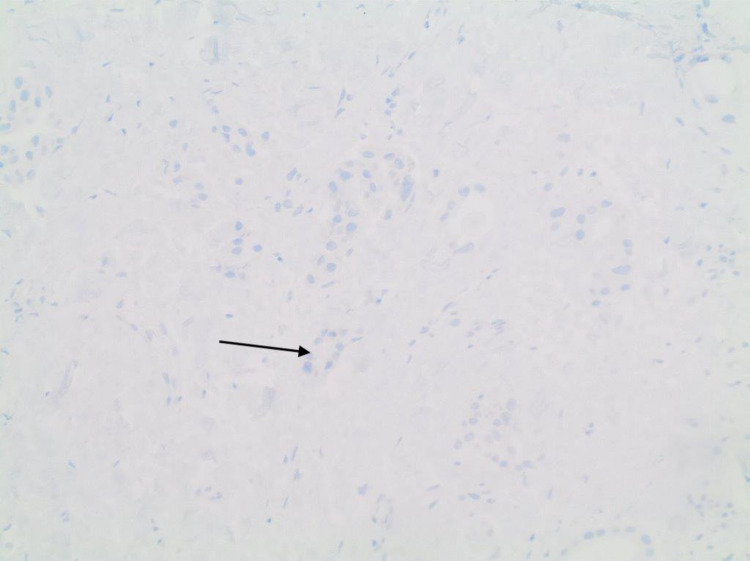
Negative immunostaining for CK20. CK20: cytokeratin 20

A 61-year-old gentleman with a past medical history of diverticulitis status post partial colectomy 10 years earlier and lumbar spinal fusion presented to the outpatient clinic with painless lower right eyelid swelling that slowly increased over a four-month duration. Associated symptoms included a watery right eye for a few months but no headaches or vision changes. His family history was significant for breast cancer in the mother. The patient was a lifetime nonsmoker with no hazardous occupational history. Physical examination was only positive for a firm internal mass stretching across the width of the right lower eyelid with intact overlying skin. No erythema or ulceration was noted. Extraocular muscle movements were noted to be intact. Pupils were small, equal, and reactive bilaterally. Neck examination was significant for palpable enlarged upper right cervical lymph node about 1.5 cm and a smaller <1 cm lymph node posterior to that. CT of the orbit showed a 4.5 cm × 1.3 cm ill-defined soft tissue mass of the lower medial wall of the right orbit with the destruction of the medial wall of the orbit and extending into right ethmoid air cells with no involvement of the optic nerve. Incision biopsy of the mass showed an adenocarcinoma (Figures 1, 2) that was positive for cytokeratin (CK)7 staining (Figure 3). Immunostain for thyroid transcription factor 1 (TTF-1) (Figure 4), S100, and CK20 (Figure 5) was negative.

**Figure 6:**
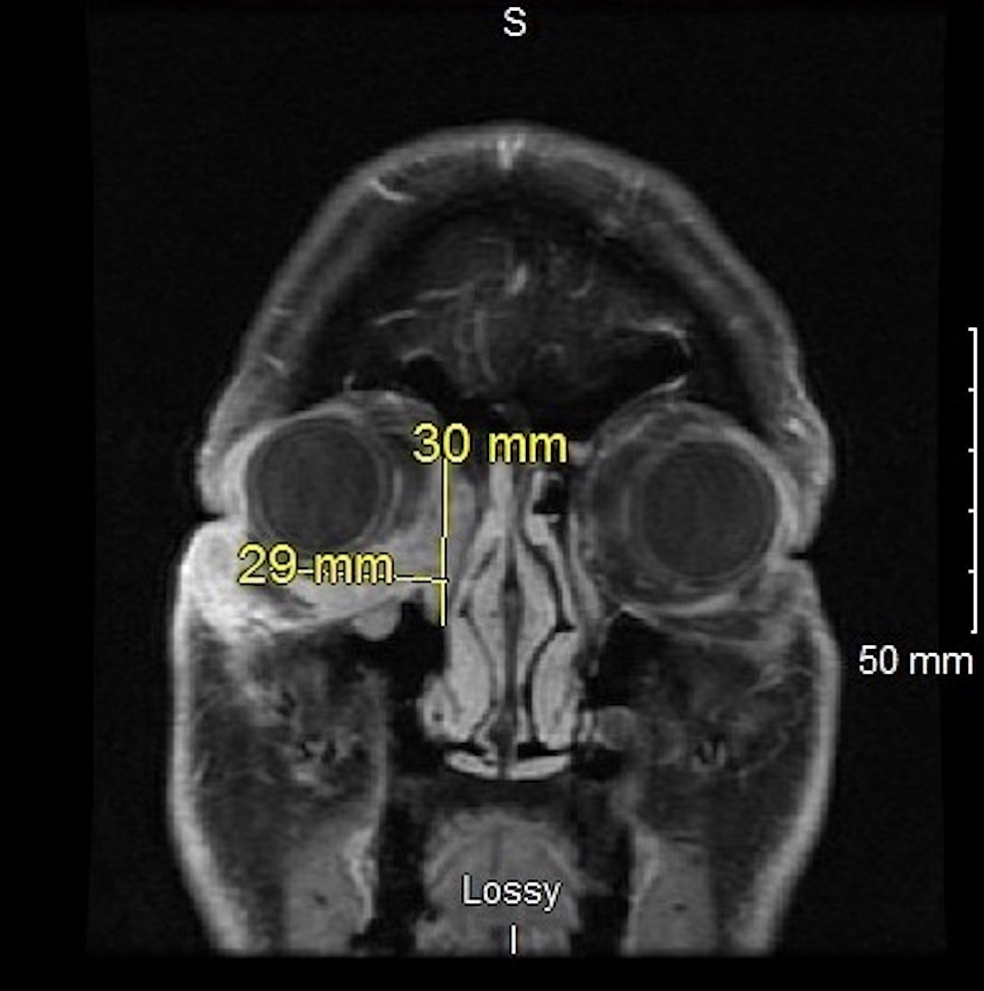
Head MRI coronal section showing the infiltrative mass extending from the inferior right periorbital region to the medial canthus. MRI: magnetic resonance imaging

**Figure 7:**
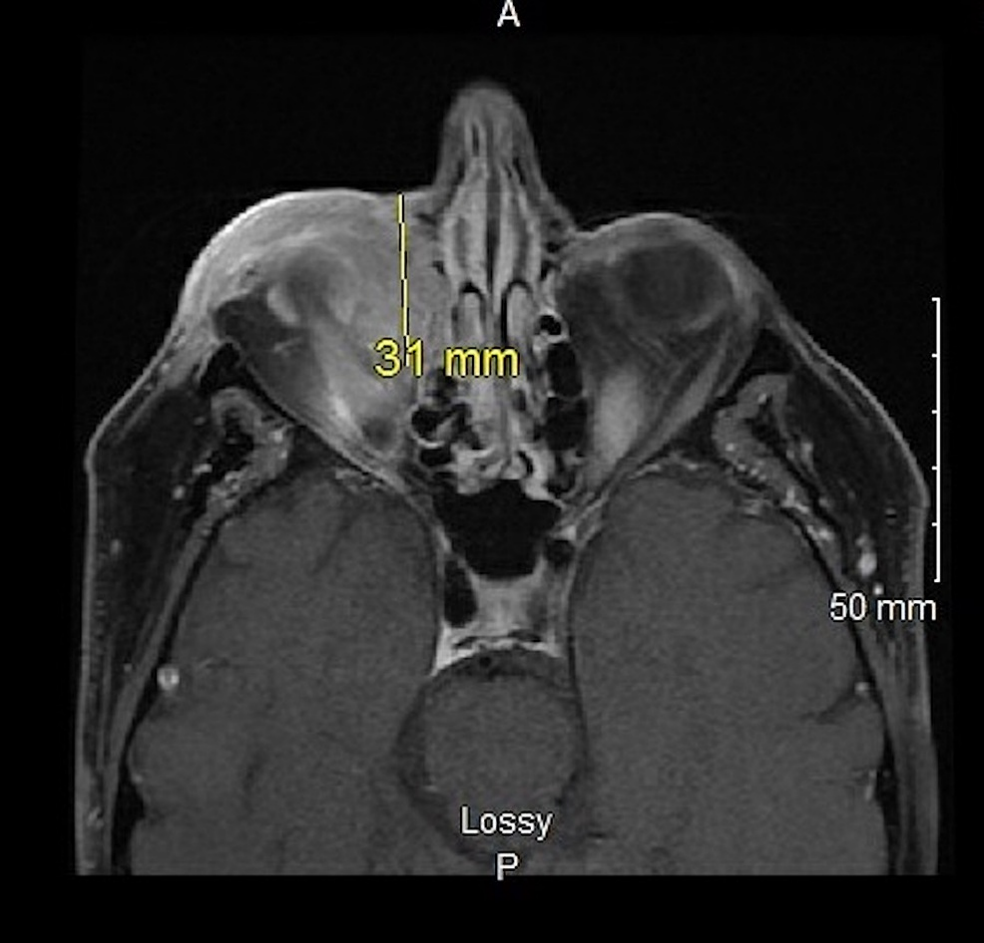
Cranial MRI axial section showing the infiltrating right orbit tumor. MRI: magnetic resonance imaging

CT scans of the chest/abdomen/pelvis with and without contrast were negative for evidence of any chest malignancy. No intra-abdominal or hepatic masses, bone lesions, or intra-abdominal adenopathy were observed. Positron emission tomography/computed tomography (PET/CT) showed positive activity in the lower eyelid region and slight activity in the right submandibular nodal region. MRI of the orbits was significant for an enhancing infiltrative mass extending from the inferior right periorbital region to the medial canthus and posteriorly into the orbit, as well as the right ethmoid sinuses. The mass was infiltrating the extraconal fat within the orbit but without infiltration of the right optic nerve or retrobulbar fat (Figures 6, 7).

The patient was transferred to a tertiary care center where he was scheduled for a right eye enucleation and right neck node sample. Due to extensive local invasion of the tumor, the patient underwent an extensive surgery that included right maxillectomy with orbital exenteration, right total ethmoidectomy, right sphenoidotomy, right frontal sinusotomy with the removal of the anterior table of the frontal sinus, skull base resection, and right selective neck dissection levels 1 through 4. The patient underwent a reconstruction of the skull base. Operative biopsy was conclusive of a high-grade apocrine adenocarcinoma (3.7 cm) extensively involving the infraorbital dermis and subcutaneous tissue, sinonasal mucosa (both maxillary and ethmoid sinuses), periorbital soft tissue with extensive lymphatic invasion including dermal lymphatics, optic nerve, and bone margins were negative. Selective neck dissection was positive for a metastatic adenocarcinoma (3.5 cm) in one of the right jugulodigastric nodes, metastatic adenocarcinoma (2.5 cm) in two of the level 1B nodes. Metastatic adenocarcinoma of right neck level 3 (0.5 cm) in two nodes, level 2 (1.1 cm in six of twelve nodes and level 4 dissection (1.1) in twelve of twenty-four lymph nodes. The skull base margin was also invaded by malignant cells. Given the aggressive tumor pathology and natural history, the patient was a candidate for adjuvant radiotherapy. He received intensity-modulated radiotherapy (IMRT) to maximize the coverage of the target tissues and minimize dose to the surrounding critical structure. Daily cone-beam computed tomography (CT) was used to ensure accurate daily localization. Given several high-risk factors including positive margin, several metastatic lymph nodes, and extensive lymphatic invasion, the patient was started on weekly cisplatin. The patient completed a course of 66 Gy in 33 fractions of adjuvant IMRT along with six cycles of cisplatin. He is scheduled for a follow-up MRI of the skull base and PET/CT in three months.

## Discussion

First described in 1952 by Lennox et al., primary adenocarcinoma of the eyelid, also known as mucinous adenocarcinoma of the eyelid, is a rare type of cancer that usually arises in the periocular region [5]. The median age of onset is reported to be 60 years [6]. Previous research has suggested that these carcinomas have a predilection for females over males, with a female-to-male incidence ratio of 3:1; however, recent studies do not support this claim as case studies show higher incidence in Caucasian males. Recurrence after surgery is estimated to occur in up to 40% of cases [7]. In one study that involved 21 cases diagnosed with mucinous sweat gland adenocarcinoma of the eyelid, 40% of patients had one or more local recurrences, one patient died due to extensive local invasion of facial tissue after multiple recurrences, and one patient had submandibular lymph node metastasis that was treated by radical neck dissection [4].

These lesions develop insidiously without pain and can easily be mistaken as benign lesions. For this reason, patients tend to present with a prolonged course of an “uncomfortable” nodular bump on the eyelids. These lesions have a vast array of colors ranging from flesh-colored to tan, gray, or blue. They appear smooth, bumpy, and tend to have a firm nodular pedunculated or papillomatous appearance [8]. Upon visual inspection, the variation in the appearance of these lesions enables them to mimic basal cell carcinoma, cysts, chalazion, and keratoacanthoma [9]. Clinical diagnosis can sometimes be challenging because these lesions often look like other pathologies. Therefore, a biopsy is required to confirm the diagnosis of any suspected lesion. Previous research has suggested that these lesions are eccrine; however, recent studies have shown that malignancy affects both apocrine and eccrine glands. While eccrine sweat glands exist in different parts of the skin, apocrine sweat glands exist in relatively hairy regions of the body, mainly in the axilla and around the nipple, the perianal, and the perineum regions. The ceruminous gland is present in the external auditory canal and Moll’s gland in the eyelid. Histologically, this neoplasm is composed of cuboidal basal cells surrounded by stromal mucinous invasion, low mitotic count, and little nuclear atypia [10].

Immunohistochemistry plays a crucial role in diagnosis as the exclusion of the tumor as a metastasis from other primary sites is essential for proper diagnosis. Primary mucinous carcinoma tumor cells are CK7-positive and CK20-negative. The expression of p63 by these tumors and its diagnostic accuracy are still controversial [11].

Primary adenocarcinoma of the eyelid is a low-grade malignant tumor with the ability to invade locally. It rarely metastasizes to lymph nodes and distant sites [12]. This makes our case unique as the patient had proven metastasis to cervical lymph nodes along with the extensive local invasion of surrounding tissues. Given the disease rarity, there is no standard of care for the surgical treatment of PMA. Currently employed modes of treatment include standard excision to wide local excision with dissection of regional lymph nodes; however, the location of these neoplasms often necessitates narrow margins with reconstruction based on the tumor’s stage [13]. Although Mohs micrographic surgery is a surgical option, it is a less commonly used procedure, with only 9.4% of cases estimated to be treated with Mohs micrographic surgery. Orbit exenteration and adjuvant radiation therapy have been reported by some authors [14]. On the other hand, adjuvant chemotherapy has been deemed to be indicated in cases with lymph node metastasis. Suggested chemotherapy regimens include cisplatin, cyclophosphamide, and Adriamycin [15]. In contrast to basal and squamous cell carcinomas, PMA tumors located on the head and neck are more likely to have a better prognosis and a lower rate of recurrence. Young age at presentation, caucasian race, positive surgical margin (incomplete resection of the tumor), and tumors >1.7 cm at presentation have been reported to be poor prognostic factors associated with a higher recurrence rate [16]. Given the paucity of data available to date, there is no definite data to guide postoperative systemic treatment and follow-up.

## Conclusions

PMA is a rare malignancy that usually exhibits low-grade features and presents as a slowly enlarging mass. Histopathology and immunohistochemistry are the cornerstones of diagnosis. Given the disease rarity, there is a lack of sufficient data to guide management and long-term follow-up. This case should raise awareness of the importance of evaluating eyelids as part of physical examination and reminds us that not every eyelid induration is from a chalazion. Primary care physicians, surgeons, and ophthalmologists should be aware of this tumor and consider it in the differential diagnosis of solid eyelid lesions. Early diagnosis and treatment can save the eye.
